# On Amaurosis in Albuminuria, and Hyperacusia in Facial Paralysis

**Published:** 1851-10

**Authors:** 


*On Amaurosis in Albuminuria, and Hyperacusia in Facial Paralysis.* By M. LANDOUZY.—M. Landouzy has recently made additional communications upon these subjects to the Academie. In regard to the occurrence of *disturbance of vision in albuminous nephritis,* his first publication took place in 1849, and although some observers have since failed to detect this coincidence, several others, among whom are MM. Roux, Forget, and Cunier, have been more successful; and in this Second Memoir, the author believes himself fully authorized in concluding:—1. That disturbance of vision is almost a constant symptom of albuminous nephritis, constituting a new species of amaurosis, which may be termed *albuminuric.* 2. The amaurosis is not attributable to a deterioaation of strength. 3. It very frequently is the initial symptom, appearing before other pathognomic signs. 4. It appears, disappears, and returns, without exactly corresponding to the phases of the albuminuria or the œdema. 5. It leads to the belief that albuminous nephritis is the result of a change in the ganglionic system. In a still more recent communivation (January 28) M. Landouzy refers to additional confirmatory observations, and states that he has now had frequent opportunities of remarking the amaurosis in albuminuria, consequent upon the application of two large blisters.

*Excessive sensibility of the organ of hearing in facial paralysis* is another of the unobserved coincidences to which M. Landouzy claims the merit of drawing attention. It is true that such was observed by M. Roux in his own case many years since, but was then considered as a mere accidental anomaly. M. Landouzy has now collected several cases in which hyperacusia existed, and from a consideration of these, draws the following conclusions:—1. Hyperacusia of the paralysed side is an almost constant symptom in facial hemiplegia, when independent of all cerebral affection. 2. It appears at the same time as the paralysis, and disappears before this. 3. It may be attributed to paralysis of the *tensor tympani.* 4. Hyperacusia may be present in some cases without facial paralysis. 5. Whether coinciding with the hemiplegia, or existing independently, it disappears spontaneously and completely, in a space of time varying from a fortnight to three months. 6. In some cases, in order to verify its existence, it is necessary to impress the ear with a loud, sudden sound, and the more so the longer the affection has continued. 7. Treatment directed especially to this symptom almost always fails to procure relief.—*Ibid, from Gaz. Med.,* 1850.

